# Diffusion kinetics and perfusion times in tissue models obtained by bioorthogonal Raman *μ*-spectroscopy

**DOI:** 10.1016/j.bpr.2024.100150

**Published:** 2024-03-05

**Authors:** Saskia Altmaier, Ina Meiser, Frank Stracke, Heiko Zimmermann

**Affiliations:** 1 Department of Molecular and Cellular Biotechnology, Saarland University, Saarbruecken, Germany; 2 Fraunhofer Institute for Biomedical Engineering (IBMT), Joseph-von-Fraunhofer-Weg 1, Sulzbach, Germany; 3 Facultad de Ciencias Del Mar, Universidad Católica Del Norte, Coquimbo, Chile

## Abstract

The penetration kinetics of small-molecule compounds like nutrients, drugs, and cryoprotective agents into artificial cell aggregates are of pivotal relevance in many applications, from stem cell differentiation and drug screening through to cryopreservation. Depending on compound and tissue properties as well as aggregate size and shape, the penetration behavior can differ vastly. Here, we introduce bioorthogonal Raman microspectroscopy as a contactless technique to investigate the penetration of various compounds into spheroids, organoids, and other tissue models in terms of diffusion coefficients and perfusion times. We showcase the potential of the method by applying it to the radial perfusion of neural stem cell spheroids with the prevalent cryopreservation additive dimethyl sulfoxide. Employing a diffusion model for spherical bodies, the spectroscopic data were quantitatively analyzed. Perfusion times were obtained for spheroids in the sub-mm region, and interesting findings about the spheroid-size dependence of the diffusion coefficient are reported.

## Why it matters

Organ and tissue models are increasingly important to modern pharmaceutical screening, and due to the often extended time periods essential for differentiation, cryopreserved storage is a mandatory requirement. Unfortunately, cryopreservation of functional 3D cell aggregates is still far from simple. A major reason is the lack of knowledge about the transport properties of compounds like water and cryoprotective agents inside a tissue. In particular, appropriate incubation times of 3D tissue models in cryoprotectant solutions may only be derived based on accurate perfusion times. Such perfusion times may vary drastically and depend on compound properties, aggregate diameter and porosity, cell type(s), and temperature. Too short an incubation time will harm the tissue model due to internal ice formation, while too long an incubation will exert toxic effects.

## Introduction

Diffusion kinetics in biological tissues have been investigated for decades (1,2,3,4,5,6,7,8). Quantification of such transport phenomena is pivotal to pharmacology (9,10,11), cryopreservation (12,13,14), metabolism and cell signaling studies (1,2,3,5,6), food science (15,16), sample preparation (11), and medical diagnostics (17,18,19). On the macroscopic scale, magnetic resonance imaging (20,21), attenuated total reflection spectroscopy (14,22), and permeation into acceptor reservoirs (6,9) are the predominant analytical techniques. Optical methods prevail in diffusion studies on sub-mm tissue samples. Fluorescent compounds are traced easily in tissues (3,4,23,24) using laser scanning microscopy. Nonfluorescent substances require fluorescent labeling (altering the diffusion and partition properties), use of fluorescent phantom compounds (having similar physical and chemical properties), or a probing modality beyond fluorescence, like Raman scattering (25,26,27). Raman techniques applied to biological samples often suffer from considerable unspecific backgrounds. This may be circumvented by minor chemical alterations of the investigated compound, which leave diffusion and partition properties almost unchanged while causing spectroscopic separation from the unspecific background. Such experimental strategies are frequently called bioorthogonal labeling (28). In this work, we demonstrate a tailored optical technique for the determination of diffusion coefficients and perfusion times of exogenous compounds into sub-mm 3D cell aggregates like spheroids, embryoid bodies, organoids, or other tissue models using bioorthogonal Raman microspectroscopy.

The generation and application of such 3D cell aggregates have become fundamental in biomedical research (29,30,31,32) and in pharmaceutical screening (33,34,35). They provide unique prospects as preclinical model systems to investigate human physiology and to accelerate diagnostics as well as drug development. Due to widespread fields of applications, the impact and demand for ready-to use 3D cell aggregates has steadily increased in the last decade. However, the differentiation process of these systems is often complex, time consuming, and technically challenging. Thus, suitable cryopreservation routines as a missing key technology to enable long-term cryostorage of relevant 3D model systems have to be established (36). One of the main hurdles for the cryopreservation of tissue models is the retarded penetration of cryoprotective agent (CPA). The prerequisite for successful cryopreservation is a sufficient level of CPA throughout the system while avoiding cytotoxic effects. Both cryoinjury in unprotected cells and cytotoxicity of CPA endanger the cell integrity and the associated viability and functions of the tissue (37). Accordingly, we introduced bioorthogonal Raman microspectroscopy to determine the perfusion times of CPA into 3D cell aggregates. Knowledge of perfusion times is the key to appropriate compound application and tissue cryopreservation (12,38). In particular, we investigated the penetration of deuterated dimethyl sulfoxide (DMSO-d6) as the most common and potent permeable CPA. Neural stem cell (NSC) spheroids of different sizes and ages serve as homogeneous tissue models. The introduced method is versatile and may be easily translated to other 3D model systems and compounds. The potential limitations and possible enhancements of the technique are discussed by means of the obtained results.

## Material and methods

### NSC culture

The human induced pluripotent stem cell line UKKi011-A (https://ebisc.org/UKKi011-A) was differentiated into NSCs using a modified protocol based on Reinhardt et al. (39). The cells were maintained on tissue culture-treated 6 wells and precoated with 9 *μ*g/cm^2^ human embryonic stem cell qualified Matrigel (Corning, Corning, NY, USA) with NSC medium. The medium consists of DMEM/F12 without L-Glutamine and HEPES (–/–) and neurobasal medium (1:1), 0.5 mg/mL Pen/Strep/L-Glut, 1% B27 supplement, 0.5% N2 supplement, 225 *μ*M L-AA (all Fisher Scientific, Schwerte, Germany), 3 *μ*M CHIRS 99021, 0.6 *μ*M PMA (Bio-Techne, Minneapolis, MN, USA). The cells were cultivated at 37°C and 5% CO_2_ for 5–7 days before passaging, and the medium was changed every 48 h.

### Generation of NSC spheroids

For splitting, NSCs were incubated with 1 mL prewarmed Accutase solution (Fisher Scientific) for 5 min at 37°C. The cell monolayer was thoroughly rinsed with 3 mL DMEM/F12 GlutaMAX to stop the reaction and resuspend the detached cells. After centrifugation at 300 × *g* for 3 min, the supernatant was discarded and the pellet resuspended in fresh NSC medium. To generate different spheroid sizes, NSCs were seeded with different initial cell concentrations (2500 cells/well and 12,000 cells/well) in 96-well Ultra-Low-Attachment plates (Fisher Scientific). The spheroids were cultivated for 3, 7, or 14 days at 37°C and 5% CO_2_ to assess the influence of different spheroid sizes and ages. Medium was carefully changed every 48 h.

### Two-photon excitation microscopy

**Figure 1:**
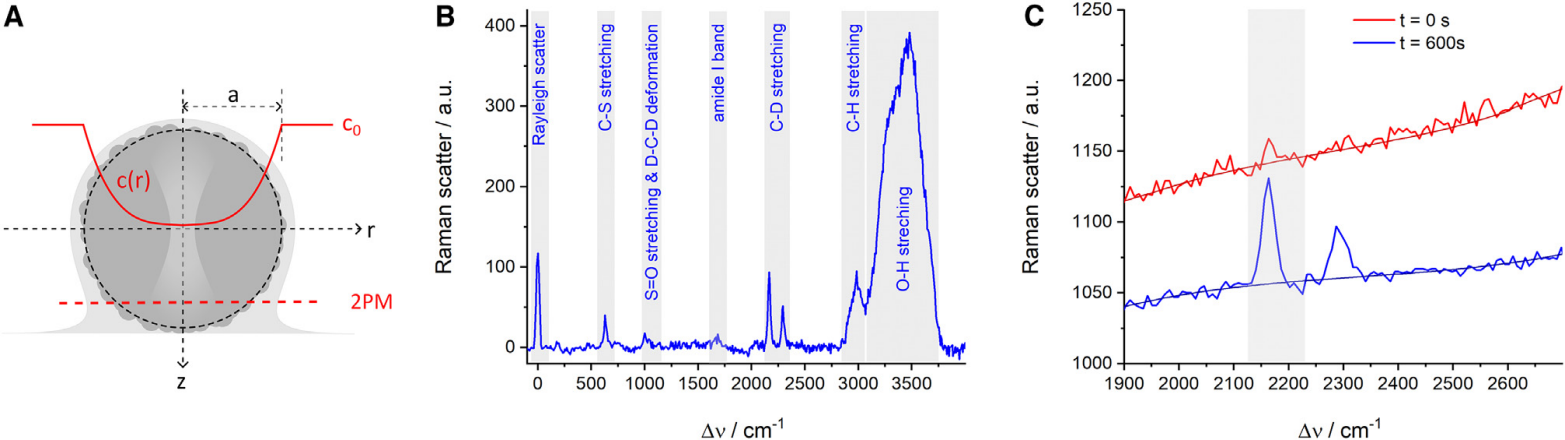
Schematic of experimental procedures. (*A*) The center of a spherical tissue model immersed in a medium drop (containing DMSO-d6 at a concentration of *c*_*0*_) is repeatedly probed for DMSO-d6 concentration *c(0,t)* by confocal Raman microspectroscopy. Two-photon micrographs were recorded 50 *μ*m below the spheroid surface. (*B*) Typical background-corrected Raman spectrum of a NSC spheroid center and the respective vibrational band assignments. (*C*) Sections of an early (t = 0 s) and a late spectrum (t = 600 s) from a DMSO-d6 penetration experiment. The panel shows the background generation using a B-spline fit as well as the band integration limits for DMSO-d6 quantification (highlighted in grey).

For two-photon excitation microscopy, viable spheroids were stained with 100 mM Rhodamin B for 24 h and NucBlue Live ReadyProbes Reagent (Hoechst 33342) (Fisher Scientific) for 5 h at 37°C and 5% CO_2_ to visualize membrane structures and cell nuclei. To detect intercellular spaces, spheroids were incubated with 10 mM fluorescein (Fisher Scientific) for 1 h. Two-photon micrographs were recorded 50 *μ*m below the spheroid surface as indicated in Fig. 1 *A* using a Trimscope Matrix operated at an excitation wavelength of 780 nm (Miltenyi, Bergisch-Gladbach, Germany).

### Raman acquisition protocol

To quantify DMSO penetration into NSC spheroids, the temporal progression of DMSO concentration at the spheroid center is recorded by means of confocal Raman microspectroscopy. A detailed description of the technical setup can be found elsewhere (40,41). A Nikon Plan Apo 20×/0.75 objective was used for focused illumination and Raman scatter collection. The illumination wavelength was λ_0_ = 532 nm. Using a 300 mm^−1^ grating, we find a spectral resolution of about 38 cm^−1^.

For the quantification of DMSO, the most intense band in the DMSO Raman spectrum was used, originating from the CH-stretching vibration. Since this band spectrally coincides with analog vibrations of many other CH-containing compounds of the tissue, DMSO was bioorthogonally labeled by replacing the hydrogen atoms (*M* = 1 g/mol) with deuterium atoms (*M* = 2 g/mol). DMSO (*M* = 78 g/mol) and deuterated DMSO-d6 (*M* = 84 g/mol, AppliChem GmbH, Darmstadt, Germany) behave almost identically in diffusion and partition processes while the major vibration band (CH) is shifted from about 2900 to 2160 cm^−1^ (CD) (26), as shown in Fig. 1 *B*. The spectral region around 2000 cm^−1^ is almost free of interfering bands and can be analyzed easily.

A crucial task in sample preparation is spheroid immobilization. It has to be attached to the substrate strongly enough to withstand shear forces due to the medium exchange and to hold its position during the acquisition. At the same time, the contact area between spheroid and substrate must be as small as possible since a uniform influx of DMSO from all sides and a spherical geometry of the tissue model are prerequisites for the data analysis herein. To this end, 15 *μ*L drops of 20% Poly-L-Lysine in PBS without calcium and magnesium (–/–) were spotted onto a microscopy slide and incubated for 30 min at 37°C. Successively, the supernatant solution was discarded and the slide dried at room temperature. The spheroids were incubated on the dry spots with 15 *μ*L medium drop for 4 h at 37°C and 5% CO_2_ to attach on to the glass surface.

The immobilized spheroids were positioned on the microscope to their maximum cross section in the focal plane (equatorial *z* adjustment) and to the laser focus at the center of that cross section (*xy* adjustment), as shown in Fig. 1 *A*. 15 *μ*L DMSO-d6 (20%) in PBS (–/–) was gently added by a micropipette, resulting in a surrounding concentration *c*_*0*_ of 10%. The acquisition was started about 20–30 s post-DMSO-d6 addition as a sequence of 600 Raman spectra of 1 s exposure time each. The varying dead time between addition and acquisition start is accounted for as *t*_*0*_ in the fit model.

### Data processing

Data processing was performed using OriginPro 2020 software (OriginLab Corp., Northampton, MA, USA). First, each spectrum *i(Δν)* of a sequence was background corrected by subtracting a baseline *b(Δν)* generated by seven-point B-spline interpolation in the spectral area shown in Fig. 1 *C*. The Raman band integral was calculated from *ν*_*0*_ = 2124 to *ν*_*1*_ = 2233 cm^−1^ (highlighted in Fig. 1 *C*).

(1) $$A\left(t\right)=\int_{\nu_{0}}^{\nu_{1}}\left[i\left(\Delta \nu \right)-b\left(\Delta \nu \right)\right]d\Delta \nu$$

The Raman band integral *A(t)* is considered to be proportional to the DMSO concentration at the spheroid center *c(0,t)*. In order to analyze the concentration data, a fit equation was derived from an expression for the uniform diffusion of a compound from an infinite reservoir at *c*_*0*_ into a sphere (42). The general differential equation of diffusion is solved for the given geometry to yield a Laplace series expansion. For the fit function, the series is aborted at *n* = 8. Higher elements only contribute significantly to the very early range of the sigmoidal function where no data were recorded anyway (*t* < *t*_*0*_).

(2) $$\frac{c\left(0,t\right)}{c_{0}}=\frac{A\left(t\right)}{A_{0}}=1+2\sum_{n=1}^{8}{\left(-1\right)}^{n}\;\exp\left[-Dn^{2}\frac{\pi^{2}}{a^{2}}\left(t-t_{0}\right)\right]$$

**Figure 2:**
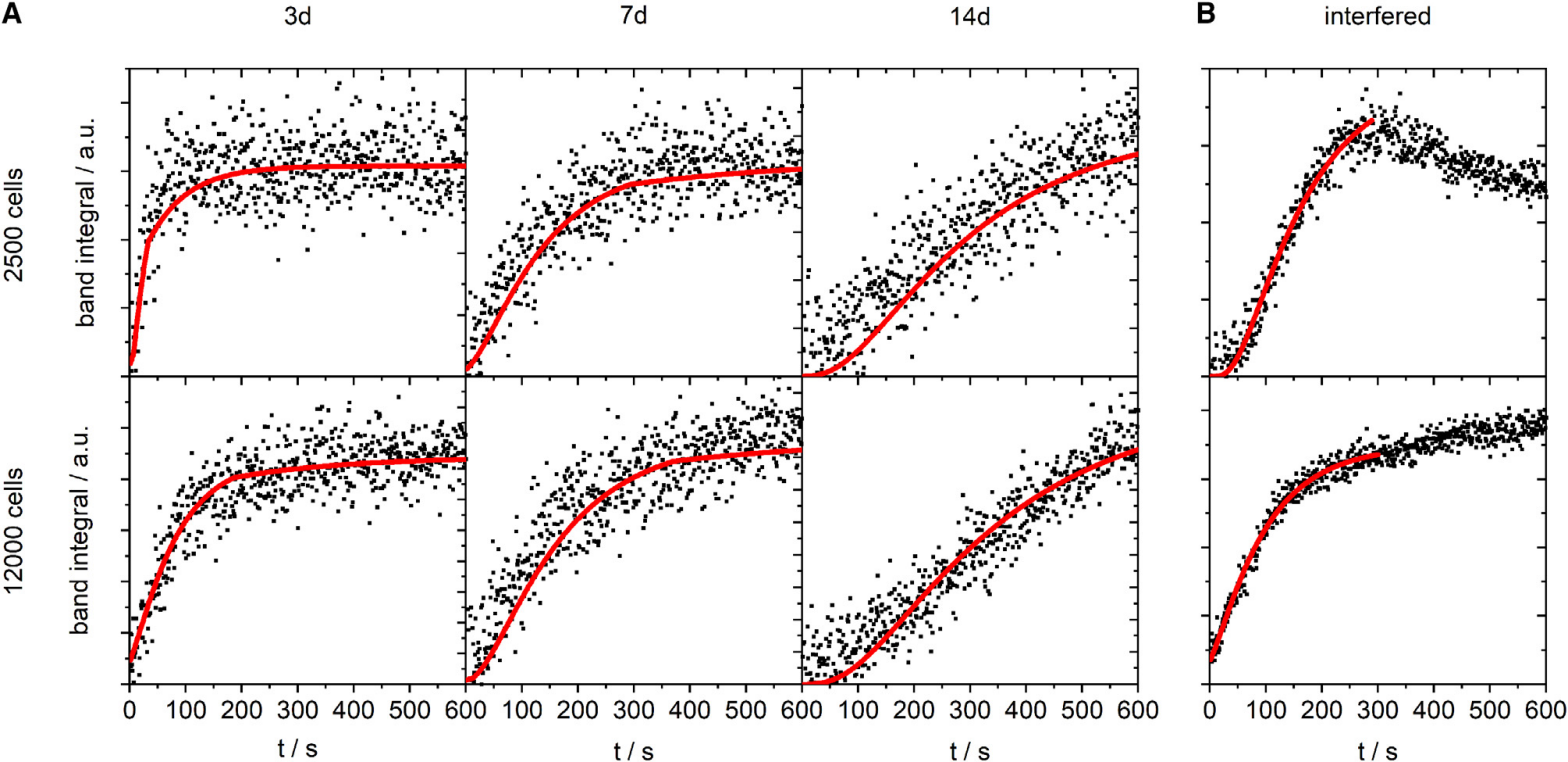
DMSO-d6 perfusion courses for spheroids of different initial cell number (and hence initial size) and age. Each data point *A(t)* was derived from a Raman spectrum according to Eq. 1. All shown example data sets in (*A*) are essentially undisturbed by external effects. Red curves are fits of Eq. 2 to the data yielding Dπ^2^/a^2^ as fit parameter. (*B*) displays examples of interfered data sets with partial fits.

The spheroids are assumed spherical, and their mean radii *a* were determined from micrographs in order to derive the averaged diffusion coefficient *D* of the respective spheroid tissue from the fitting parameters. Fig. 2 displays example data for each condition and the respective fit curves. About 2/3 of the spectral sequences show interferences due to external influences as medium convection or spheroid drift. These disturbed data sets were either discarded from analysis or analyzed in the marginally disturbed ranges only.

## Results

The bioorthogonal exchange of hydrogen against deuterium shifts the most prominent vibration bands of DMSO-d6 into a spectral range with almost no interference. The B-spline on the spectral data reliably provides suitable baselines. Quantification of DMSO-d6 at the spheroid center by integration of the spectral band as indicated in Fig. 1 *C* led to the expected sigmoidal increase (Fig. 2). The early part of the sigmoid with positive curvature is often invisible for small tissue models with fast influx since it is passed through during the dead time t_0_.

A significant fraction of the acquired influx data was interfered by external effects such as sample drift, shrinking and swelling or medium convection. As long as only parts of a data set were affected, the undisturbed data were fitted to yield diffusion parameters. All parameters from plausible fits were included into the analysis. If a data set was interfered throughout or could not plausibly be described by the model, it was discarded from the analysis (this applied to 11 out of 94 runs in total).

In order to evaluate the dependence between DMSO diffusion behavior and size as well as age of the tissue model, two characteristics are examined. First, the diffusion coefficient *D* as a tissue-specific measure is regarded. Obviously, *D* is an averaged value over the spheroid volume herein. Secondly, the perfusion time as a spheroid-specific measure is analyzed. We will define the perfusion time as the time the relative concentration *c/c*_*0*_ = *A/A*_*0*_ at the spheroid center reaches 90%. At such a late stage of the process, the sigmoidal curve is dominated by the first element (*n* = 1) of the Laplace expansion, and the perfusion time becomes:

(3) $$t_{90\%}=-\frac{a^{2}}{D\pi^{2}}\cdot \ln\frac{1}{2}\left(1-\frac{A}{A_{0}}\right)=\frac{\ln\;20}{D}\cdot \frac{a^{2}}{\pi^{2}}\text{.}$$

**Figure 3:**
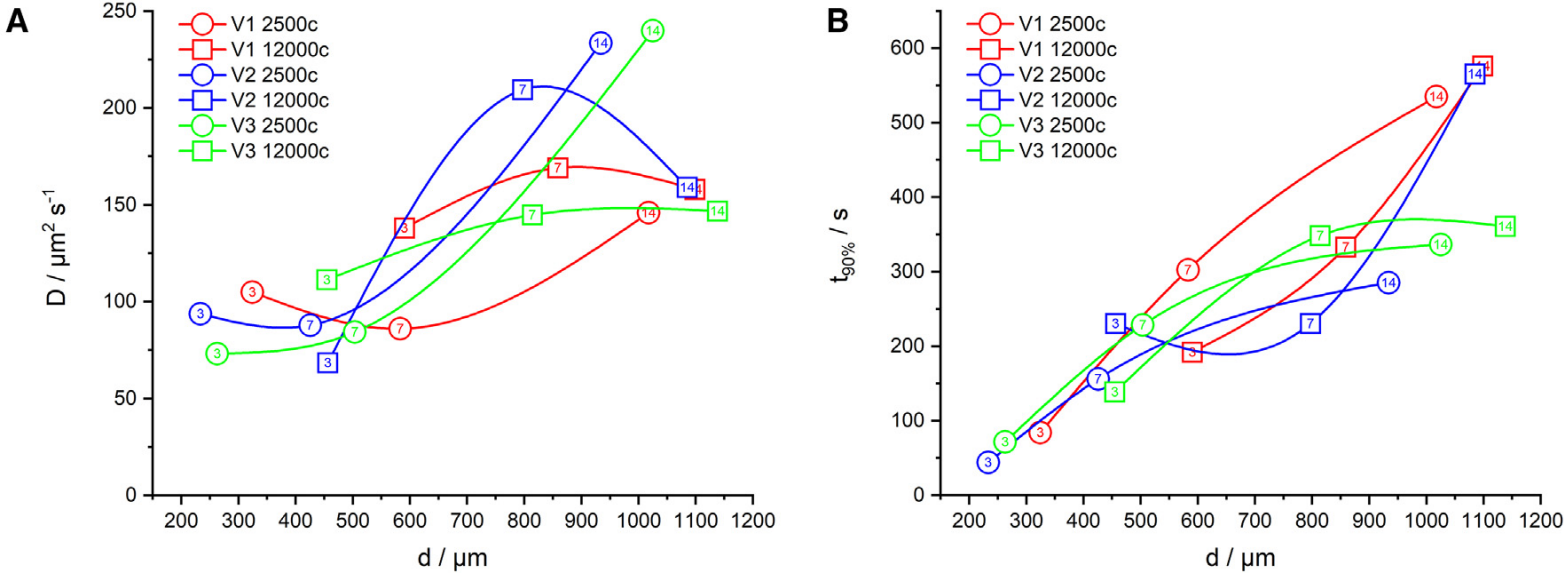
Plot of derived diffusion coefficients *D* (*A*) and perfusion times t_90%_ (*B*) against the spheroid diameter *d*. Three biological replicates (V1, V2, V3) were carried out. Each run is color coded and connected by a line. Circles represent data of runs with 2500 cells at inoculation, and boxes stand for 12,000 cells at inoculation. The ages of spheroids in days are given as numbers within the symbols (3, 7, and 14 days from left to right). Each symbol comprises 4–6 probed spheroids.

Obviously, the perfusion time acts reciprocally to the diffusion coefficient and proportional to the spheroid radius squared. Hence, assuming a fixed *D*, a plot of *t*_*90%*_ against the spheroid diameter *d* should result in a parabola. In contrast, our data show size-dependent diffusion constants (Fig. 3 *A*). Up to a diameter of 500–600 *μ*m, no significant alterations in *D* were observed for the NSC tissue models. Further growth is accompanied by a considerable increase in tissue permeability. While NSC spheroids of up to 500 *μ*m diameter exhibit diffusion coefficients on the order of *D* = 90 *μ*m^2^ s^−1^, diameters of 800 *μ*m result in *D* around 180 *μ*m^2^ s^−1^. The adaption of tissue permeability to the spheroid size leads to a more linear increase of the perfusion times with diameter (Fig. 3 *B*), not a parabolic one as expected from the theory. After a certain time at a size of *d* >600 *μ*m, the increase in tissue permeability slows down or is even inverted. As can be seen in Fig. 3 *A*, *D(d)* has a positive curvature for all spheroids seeded with 2500 cells (circles) but a negative curvature for all spheroids seeded with 12,000 cells (boxes).

However, even without the squared dependence between size and DMSO perfusion time, our results widely spread from *t*_*90%*_ = 1 min for *d* = 250 *μ*m to *t*_*90%*_ = 10 min for *d* = 1100 *μ*m.

**Figure 4:**
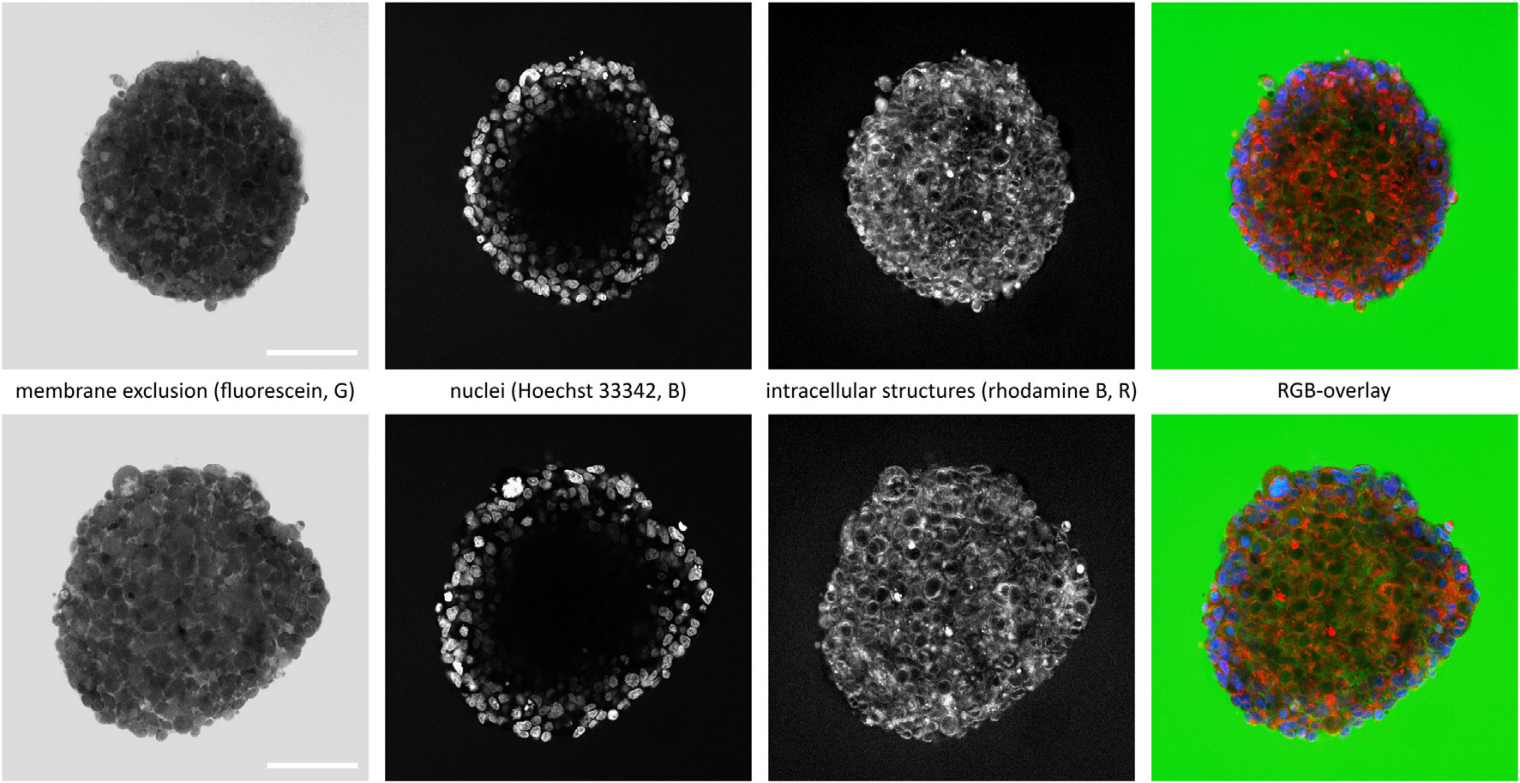
Two multichannel images of NSC spheroids acquired by two-photon excitation microscopy, image size 400 × 400 *μ*m, scale bar: 100 *μ*m. Hoechst 33342 and rhodamine B indicate cellular positions and dimensions, and membrane-impermeable fluorescein highlights extracellular voids inside the spheroids. The two-photon sections were recorded at a depth of 50 *μ*m as displayed in Fig. 1*A*.

Complementary, two-photon micrographs indicate the perfusion routes and mechanisms in a qualitative fashion (Fig. 4). The different mechanisms of tissue penetration are nicely accentuated using fluorescent dyes as mimics for negatively (fluorescein) and positively charged compounds (rhodamine B). Nonpermeant fluorescein is detected along the intercellular routes in the extracellular space. Membrane-permeant rhodamine B can be found extra- and intracellularly except in the nuclei. The nuclei of the outer cell layers of the spheroid are stained with Hoechst 33342, which is membrane permeant as well. The diffusion kinetics of Hoechst 33324 may be influenced by excessive binding to nucleic acids.

## Discussion

Raman microspectroscopy in combination with bioorthogonal labeling and tailored fit models proved to be an adequate tool for the contactless determination of compound perfusion times and averaged tissue diffusion coefficients of small-molecule compounds into tissue models. Compounds having distinct vibrational bands without interference from the spectral background can be investigated straightaway. Other compounds have to be bioorthogonally modified to introduce such a vibrational feature. The modification should not alter the diffusion and partition properties noticeably. Isotope exchange and introduction of nitrile groups are examples of possible bioorthogonal labels (28). The fit model derived herein applies nicely to the penetration of DMSO into the spherical tissue model yielding perfusion times and averaged tissue diffusion coefficients. Other sample geometries like cylinders or slabs could be analyzed with adapted fit models as well. Hence, tissue physiology, advanced drug testing, and tissue cryopreservation may considerably profit from the method.

Though there is some potential for technical improvements, the present showcase study on DMSO-d6 penetration into NSC spheroids allows some important conclusions.

First, the perfusion times t_90%_ for the pivotal CPA DMSO into sub-mm spheroids are on the order of several minutes at room temperature (Fig. 3). This proves the importance of understanding the uptake kinetics of CPA into tissues for the cryopreservation protocols of tissue models and may be relevant to adapt cell-type-, diameter-, and age-dependent DMSO incubation times prior to cryopreservation of spheroids and other 3D tissue models. The knowledge of perfusion times facilitates proper CPA incubation protocols avoiding insufficient penetration of the tissue as well as overexposure of DMSO to tissue.

Second, the perfusion time t_90%_ is strongly dependent on spheroid diameter and spreads over an order of magnitude (Fig. 3) for spheroid diameters from 300 to 1100 *μ*m. Longer perfusion times for larger spheroids are not surprising, but the strong size dependence once more proves the practical relevance of their determination.

Third, the DMSO diffusion coefficient *D* in NSC tissue supposably depends on the spheroid diameter too. Up to about *d* = 500 *μ*m, *D* seems to remain constant. Larger diameters show increasing diffusion coefficients. Since the performed experiment only probes the DMSO-d6 content in the spheroid center, we cannot discriminate between a uniform increase of *D* over the entire spheroid and a local rise of *D* just in certain layers or in the spheroid core. *D* herein is an average over the spheroid radius. So far, the cause for the increase in DMSO permeability with size is unclear. However, it was already described that spheroids >200 *μ*m diameter start to present hypoxic areas, and necrotic cores were observed in spheroids >500 *μ*m (43,44,45,46). We assume that the core of NSC spheroids with diameters >500 *μ*m also undergo oncotic necrosis due to critical shortage of oxygen. One possible cause of oncosis is the failure of the membrane pumps as a result of ATP deficiency following hypoxia resulting in increased membrane permeability (47). Thus, DMSO diffusion may be accelerated in the necrotic core of spheroids with diameters >500 *μ*m, and the average diffusion coefficient is increased. The diffusion coefficients of DMSO in NSC spheroids herein are on the same order of magnitude as values reported for DMSO in macroscopic tissues determined by osmolarity measurement (53 *μ*m^2^ s^−1^ in porcine corneoscleral discs at 0°C, (38)) or attenuated total reflection Fourier transform infrared analyses (302 *μ*m^2^ s^−1^ in porcine heart valve tissue at 22°C, (13)).

Fourth, the perfusion time of DMSO into tissue is unusually short due to its high membrane permeability. Other small-molecule compounds penetrate much more slowly, as Hoechst 33342 and rhodamine B perfusion took several hours (Fig. 4; (12)). Cell-impermeable compounds like fluorescein may still penetrate via the intercellular route (Fig. 4). Negatively charged compounds usually do not significantly permeate cell membranes, so fluorescein is predominantly found along the intercellular route, highlighting void volumes inside the tissue. Positively charged compounds like rhodamine B readily cross the cell membrane and are predominantly transported along the transcellular route. Hoechst 33342 is also highly cell permeant, but the consumption of the dye due to efficient binding in the nuclei (24) may demand a diffusion-reaction model to quantitatively describe the uptake. In conclusion, it is not possible to deduce tissue permeabilities from one compound to another. They have to be determined experimentally for each compound.

The limiting factor of the present technique turned out to be the sample handling: microspectroscopy requires long-term immobilization of the spheroid on the *μ*m scale even during fast medium exchange. The analysis using fit models for highly symmetric geometries exclude immobilization methods that occlude penetration access from any part of the surface or that deforms the sample significantly. Any attempts to mechanically immobilize the tissue models in a medium perfusion channel resulted either in excessive deformation of the sample or its breakaway during the medium exchange. The medium exchange method used herein is a provisional solution to prove the concept. Even small dislocations of the spheroid lead to signal interferences due to shifted probing locations and altered transmittances of illumination and scattered light in the tissue as observed in a significant fraction of data sets. At the moment, we are working on tissue model handling techniques allowing reliable immobilization along with fast medium switching. This will diminish most of the interfering processes observed herein and allow a defined start time. At present, the start time is blurred due to the considerable period for medium homogenization after application of the DMSO-d6 medium. However, the technology as currently available is, after all, very valuable in determining perfusion times of cell-permeant cryopreservation additives for appropriate tissue model preservation protocols.

### Outlook

The determination of perfusion times of small-molecule compounds into cell aggregates by means of bioorthogonal Raman microspectroscopy at its current status is already a valuable tool in applied cryopreservation and drug screening. For scientific purposes, more defined experimental conditions and more refined fit models are requested. Such fit models should account for the core-shell architecture of larger cell aggregates and consequently yield more than one diffusion coefficient. With respect to defined experimental conditions, the most relevant advancement will be a novel immobilization technique in combination with controlled fluidic medium switching. These techniques will provide precise starting times, homogeneous compound concentrations around the sample, and less sample drift. At the moment, we are working on different approaches to entrap spheroids and organoids stably and free from deformations. Additional temperature control will allow for more realistic studies and opens up access to Arrhenius coefficients of diffusion in tissue. Knowledge of Arrhenius parameters enables extrapolation of diffusion kinetics even into the subzero temperature regime.

Perfusion times and diffusion coefficients can be obtained for various compounds into various tissues. Compounds having salient Raman bands absent in the background may be investigated without bioorthogonal labeling. Other compounds require bioorthogonal modification like deuteration (28). Other sample geometries may be also investigated using appropriate fit models. Such models are available for 1D diffusion (from one or two planar surface(s) into a slab) and 2D diffusion into a cylinder or into a tube. As easy access to perfusion times of CPA into tissue, the technique will have a huge impact on cryopreservation of specimens beyond isolated cells. As well, it may promote proper design and interpretation of drug screening studies on 3D tissue models and organoids.

## Author contributions

S.A. performed experiments. F.S. devised spectroscopy experimental design and diffusion models and created artwork. S.A. and F.S. performed data analysis and interpretation and wrote the manuscript. I.M. devised biology experimental design, performed data interpretation, and edited the manuscript. H.Z. took over the scientific supervision and manuscript editing.
